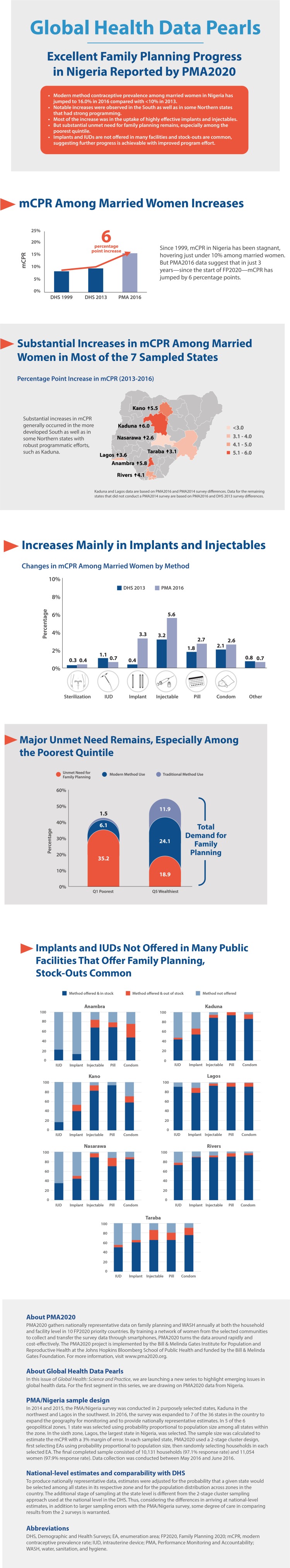# Excellent Family Planning Progress in Nigeria Reported by PMA2020

**DOI:** 10.9745/GHSP-D-17-00094

**Published:** 2017-03-24

## Abstract

- Modern method contraceptive prevalence among married women in Nigeria has jumped to 16.0% in 2016 compared with <10% in 2013.
- Notable increases were observed in the South as well as in some Northern states that had strong programming.
- Most of the increase was in the uptake of highly effective implants and injectables.
- But substantial unmet need for family planning remains, especially among the poorest quintile.
- Implants and IUDs are not offered in many facilities and stock-outs are common, suggesting further progress is achievable with improved program effort.

## Teaser

- Modern method contraceptive prevalence among married women in Nigeria has jumped to 16.0% in 2016 compared with <10% in 2013.
- Notable increases were observed in the South as well as in some Northern states that had strong programming.
- Most of the increase was in the uptake of highly effective implants.
- But substantial unmet need for family planning remains, especially among the poorest quintile.
- Implants and IUDs are not offered in many facilities and stock-outs are common, suggesting further progress is achievable with improved program effort.

**Figure:**

## mCPR Among Married Women Increases

Since 1999, mCPR in Nigeria has been stagnant, hovering just under 10% among married women. But PMA2016 data suggest that in just 3 years—since the start of FP2020—mCPR has jumped by 6 percentage points.

## Substantial Increases in mCPR Among Married Women in Most of the 7 Sampled States

### Percentage Point Increase in mCPR (2013-2016)

Substantial increases in mCPR generally occurred in the more developed South as well as in some Northern states with robust programmatic efforts, such as Kaduna.

Kaduna and Lagos data are based on PMA2016 and PMA2014 survey differences. Data for the remaining states that did not conduct a PMA2014 survey are based on PMA2016 and DHS 2013 survey differences.

## Increases Mainly in Implants and Injectables

### Changes in mCPR Among Married Women by Method

## Major Unmet Need Remains, Especially Among the Poorest Quintile

## Implants and IUDs Not Offered in Many Public Facilities That Offer Family Planning, Stock-Outs Common

### About PMA2020

PMA2020 gathers nationally representative data on family planning and WASH annually at both the household and facility level in 10 FP2020 priority countries. By training a network of women from the selected communities to collect and transfer the survey data through smartphones, PMA2020 turns the data around rapidly and cost-effectively. The PMA2020 project is implemented by the Bill & Melinda Gates Institute for Population and Reproductive Health at the Johns Hopkins Bloomberg School of Public Health and funded by the Bill & Melinda Gates Foundation. For more information, visit www.pma2020.org.

### About Global Health Data Pearls

In this issue of *Global Health: Science and Practice*, we are launching a new series to highlight emerging issues in global health data. For the first segment in this series, we are drawing on PMA2020 data from Nigeria.

### PMA/Nigeria sample design

In 2014 and 2015, the PMA/Nigeria survey was conducted in 2 purposely selected states, Kaduna in the northwest and Lagos in the southwest. In 2016, the survey was expanded to 7 of the 36 states in the country to expand the geography for monitoring and to provide nationally representative estimates. In 5 of the 6 geopolitical zones, 1 state was selected using probability proportional to population size among all states within the zone. In the sixth zone, Lagos, the largest state in Nigeria, was selected. The sample size was calculated to estimate the mCPR with a 3% margin of error. In each sampled state, PMA2020 used a 2-stage cluster design, first selecting EAs using probability proportional to population size, then randomly selecting households in each selected EA. The final completed sample consisted of 10,131 households (97.1% response rate) and 11,054 women (97.9% response rate). Data collection was conducted between May 2016 and June 2016.

### National-level estimates and comparability with DHS

To produce nationally representative data, estimates were adjusted for the probability that a given state would be selected among all states in its respective zone and for the population distribution across zones in the country. The additional stage of sampling at the state level is different from the 2-stage cluster sampling approach used at the national level in the DHS. Thus, considering the differences in arriving at national-level estimates, in addition to larger sampling errors with the PMA/Nigeria survey, some degree of care in comparing results from the 2 surveys is warranted.

### Abbreviations

DHS, Demographic and Health Surveys; EA, enumeration area; FP2020, Family Planning 2020; mCPR, modern contraceptive prevalence rate; IUD, intrauterine device; PMA, Performance Monitoring and Accountability; WASH, water, sanitation, and hygiene.
